# Morphology, ultrastructure, genomics, and phylogeny of *Euplotes vanleeuwenhoeki* sp. nov. and its ultra-reduced endosymbiont “*Candidatus* Pinguicoccus supinus” sp. nov.

**DOI:** 10.1038/s41598-020-76348-z

**Published:** 2020-11-20

**Authors:** Valentina Serra, Leandro Gammuto, Venkatamahesh Nitla, Michele Castelli, Olivia Lanzoni, Davide Sassera, Claudio Bandi, Bhagavatula Venkata Sandeep, Franco Verni, Letizia Modeo, Giulio Petroni

**Affiliations:** 1 Department of Biology, University of Pisa, Via Volta 4/6, 56126 Pisa, Italy; 2 Department of Biosciences, Romeo and Enrica Invernizzi Pediatric Research Center, University of Milan, Milan, Italy; 3 Department of Biology and Biotechnology “Lazzaro Spallanzani”, Pavia University, Pavia, Italy; 4 Department of Biotechnology, Andhra University, Visakhapatnam, India; 5 CIME, Centro Interdipartimentale di Microscopia Elettronica, Università di Pisa, Pisa, Italy; 6 CISUP, Centro per l’Integrazione della Strumentazione dell’Università di Pisa, Pisa, India

**Keywords:** Molecular evolution, Evolutionary biology, Genome, Bacteria, Microbial genetics, Taxonomy, Genome informatics, Phylogeny, Computational biology and bioinformatics, Evolution, Genetics, Microbiology

## Abstract

Taxonomy is the science of defining and naming groups of biological organisms based on shared characteristics and, more recently, on evolutionary relationships. With the birth of novel genomics/bioinformatics techniques and the increasing interest in microbiome studies, a further advance of taxonomic discipline appears not only possible but highly desirable. The present work proposes a new approach to modern taxonomy, consisting in the inclusion of novel descriptors in the organism characterization: (1) the presence of associated microorganisms (e.g.: symbionts, microbiome), (2) the mitochondrial genome of the host, (3) the symbiont genome. This approach aims to provide a deeper comprehension of the evolutionary/ecological dimensions of organisms since their very first description. Particularly interesting, are those complexes formed by the host plus associated microorganisms, that in the present study we refer to as “holobionts”. We illustrate this approach through the description of the ciliate *Euplotes vanleeuwenhoeki* sp. nov. and its bacterial endosymbiont “*Candidatus* Pinguicoccus supinus” gen. nov., sp. nov. The endosymbiont possesses an extremely reduced genome (~ 163 kbp); intriguingly, this suggests a high integration between host and symbiont.

## Introduction

Taxonomy is the science of defining and naming groups of biological organisms based on shared characteristics and, more recently, based on evolutionary relationships. Classical taxonomy was exclusively based on morphological-comparative techniques requiring a very high specialization on specific taxa. For this reason, and due to the development and rise of modern molecular tools, in the last decades this discipline has faced a significant period of crisis^1,2^. Lately, traditional taxonomy has been renewed in the so-called integrative taxonomy, which also includes ultrastructural and phylogenetic-molecular analysis^1,3^.

Now, in our opinion, new concepts and tools would allow taxonomy to make another step forward: to consider the host associated symbiont/microbiome, pursuing a further multidisciplinary integration with modern available technologies, such as bioinformatics and genomic analyses.

Indeed, although at present not mandatory for the description of an organism, the characterization of obligatory or occasional symbionts sensu de Bary, i.e. according to the definition of symbiosis as “the living together of two differently named organisms”^4^, and of its associated microbial consortium (= microbiome), could be indeed considered an important descriptor of the state of an organism, potentially influencing its development, physiology, and morphology. This opinion is in agreement with the findings of several previous studies^5–14^.

Based on the above reasons we are prone to consider the complex formed by the host plus associated microorganisms as a “holobiont”: Lynn Margulis defined the term “holobiont”^15^ as the assemblage of “two or more organisms, members of different species” (bionts), which remain associate “throughout a significant portion of the life history”^16^. Nowadays, the term holobiont is ambiguously defined; indeed, it can span from including only obligate mutualistic symbionts^17^ to somehow associated microbial consortia^18–23^. In our opinion, it would be appropriate to apply the concept of holobiont each time the association between different organisms gives rise to a functional unit in which emerging characteristics and properties are not typical of the different parts separately taken (e.g. symbiosis, mutualistic, neutral, or parasitic phenomena).

Therefore, the updated approach we are proposing implies the possibility to describe each of the organisms potentially associated to a host and, consequently, the need to build networks of complementary skills, able to combine bio-taxonomy tools, classical morphology, ultrastructure, molecular phylogeny, genomics, and bioinformatics. Hence, we propound to define this updated approach as “next generation taxonomy” (see “Discussion” section).

To exemplify the feasibility of the proposed approach we herein present the taxonomic description of the ciliate protist *Euplotes vanleeuwenhoeki* sp. nov. (Euplotia, Ciliophora) and its bacterial endosymbiont “*Candidatus* (*Ca.*) Pinguicoccus supinus” gen. nov., sp. nov. (*Opitutae, Verrucomicrobia*). Ciliates are known to form stable associations with eukaryotic^24–29^ and prokaryotic^6, 30–38^ organisms, and thus represent an ideal case of study for the proposed “next generation taxonomy”. Moreover, to the best of our knowledge, this is the first study addressing the concept of holobionts of protists in general.

In detail, we used the proposed approach combining the requirements of an integrative taxonomic description^39^, and some state of the art analyses, such as the host mitochondrial genome characterization, with the genomic study on the endosymbiont. Interestingly, we found that the endosymbiont “*Ca.* Pinguicoccus supinus” has an extremely small genome (~ 163 kbp), comparable in size to extremely reduced genomes of insect symbionts, making this bacterium the first of this category to be found in a unicellular host^40,41^. The extremely small genome size is suggestive of a high level of integration with the host, further indicating the appropriateness of the use of a unifying approach to ensure a suitable functional and taxonomical description of all the partners involved in such kind of symbioses.

## Results

### Description of* Euplotes vanleeuwenhoeki* sp. nov. (Figs. 1, 2, 3, 4 and 5, Table 1)

**Figure 1:**
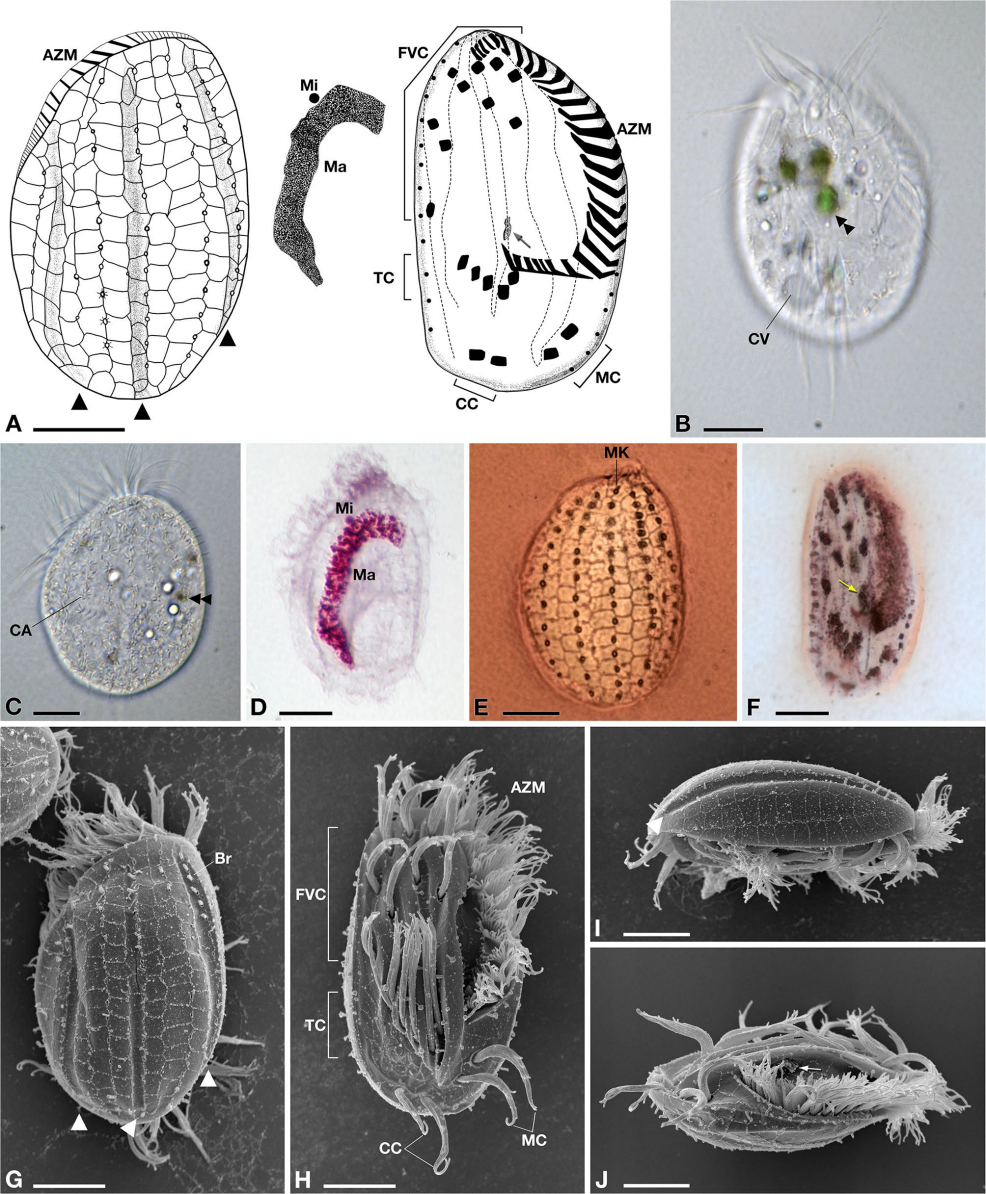
Morphology of *Euplotes vanleeuwenhoeki* sp. nov. (**A**) Schematic drawings of the dorsal side (left), ventral side (right), and nuclear apparatus (middle); (**B**) Live picture, ventral side. (**C**) Live picture, dorsal side; (**D**) Feulgen staining, showing macronucleus (Ma) and micronucleus (Mi); (**E**) Silver staining, dorsal side; (**F**) Silver staining, ventral side; (**G**–**J**) SEM pictures of dorsal side (**G**), ventral side (**H**), and lateral views (**I**,**J**); Arrow: paroral membrane; Arrowhead: dorsal furrow; Double arrowhead: food vacuole containing algae; *AZM* adoral zone of membranelles, *Br* bristle, *CA* cortical ampules, *CC* caudal cirri, *CV* contractile vacuole, *FVC* fronto-ventral cirri, *Ma* macronucleus, *MC* marginal cirri, *Mi* micronucleus, *MK* mid-dorsal kinety, *TC* transverse cirri. Bars stand for 10 µm.

**Figure 2:**
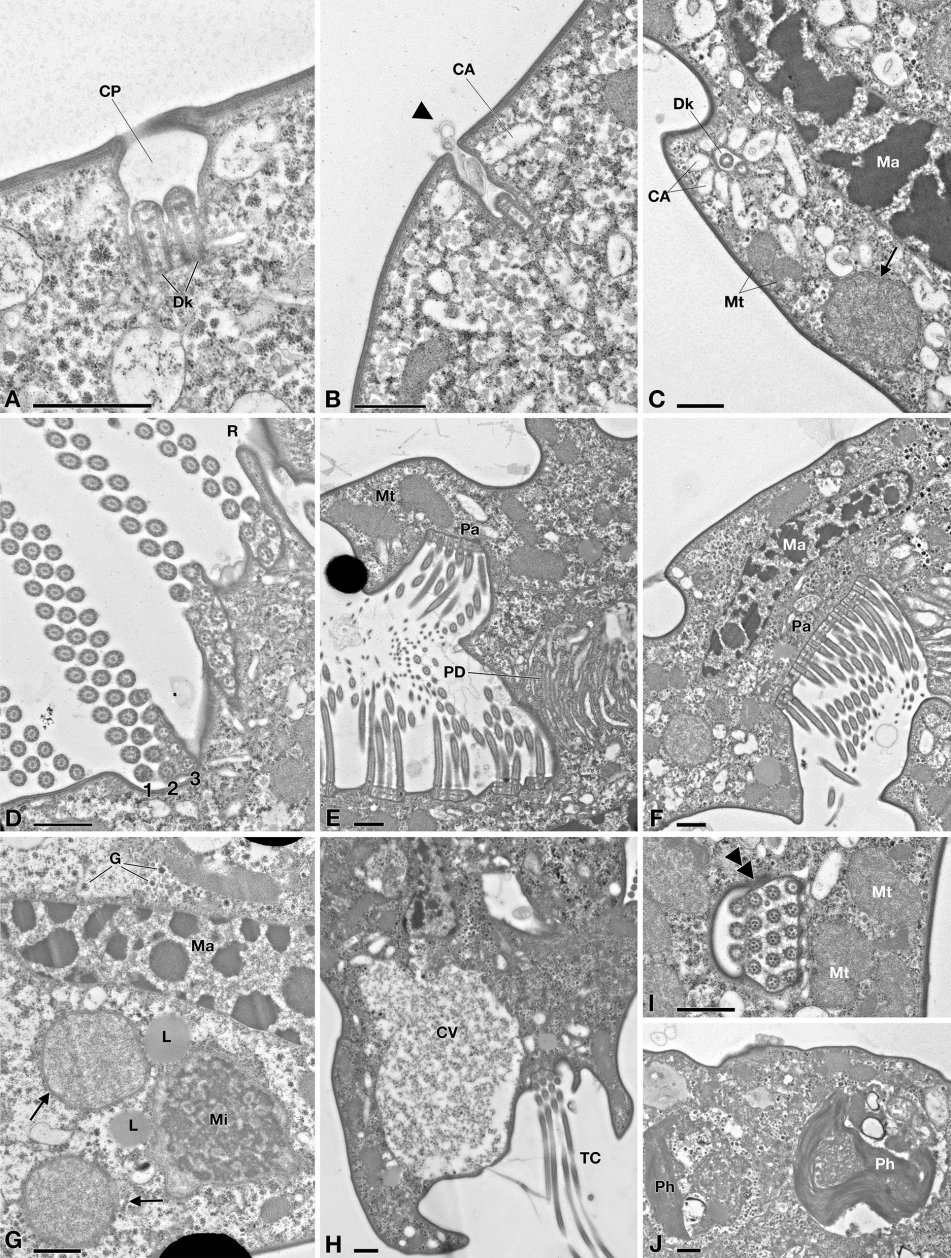
TEM picture of *Euplotes vanleeuwenhoeki* sp. nov. (**A**–**C**) Cortex region; (**D**–**F**) Oral region; (**A**) Ciliary pit (CP) with dikinetid (Dk); (**B**) Detail of bristle pit containing filamentous material (arrowhead); cortical ampules (CA) are visible; (**C**) Transverse section of dikinetid (Dk) surrounded by cortical ampules; sections of the macronucleus (Ma), two mitochondria (Mt) and an endosymbiotic bacterium (arrow) are visible; (**D**) Detail of oral membranelles, composed of two longer rows (1, 2) and one shorter row (3) of cilia; membranelles are separated by ridges (R); (**E**) Section of oral region, showing paraoral membrane (Pa) in front of oral membranelles and pharingeal disks (PD); (**F**) Closer view of paraoral membrane and transverse section of macronucleus; (**G**) Endosymbiotic bacterial cells (arrow) inside *Euplotes* cytoplasm, nearby macronucleus and micronucleus (Mi); rosettes of glycogen (G) and lipid droplets (L) are present; (**H**) Detail of contractile vacuole (CV) and transverse cirrus (TC); (**I**) Section of cirrus (double arrowhead), close to mitochondria; (**J**) Two phagosomes (Ph). *CA* cortical ampules, *CP* ciliary pit, *CV* contractile vacuole, *Dk* dikinetid, *G* rosette of glycogen, *L* lipid droplet, *Ma* macronucleus, *Mi* micronucleus, *Mt* mitochondrion, *Pa* paraoral membrane, *PD* pharingeal disk, *Ph* phagosome, *R* ridge, *TC* transverse cirrus, Arrowhead: filamentous material; Arrow: endosymbiotic bacterium; Double arrowhead: transverse section of cirrus. Bars stand for 1 µm.

**Figure 3:**
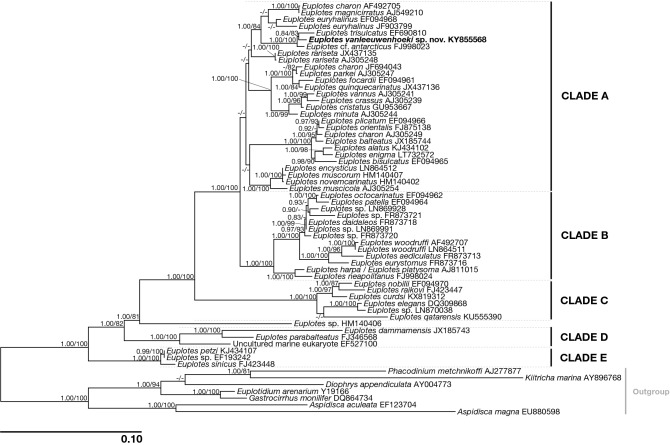
Phylogenetic tree of genus *Euplotes* based on the 18S rRNA gene. Numbers associated to nodes represent posterior probabilities and bootstrap values, respectively (only values above 0.80–75 are shown). Sequence obtained in the present work is in bold.

**Figure 4:**
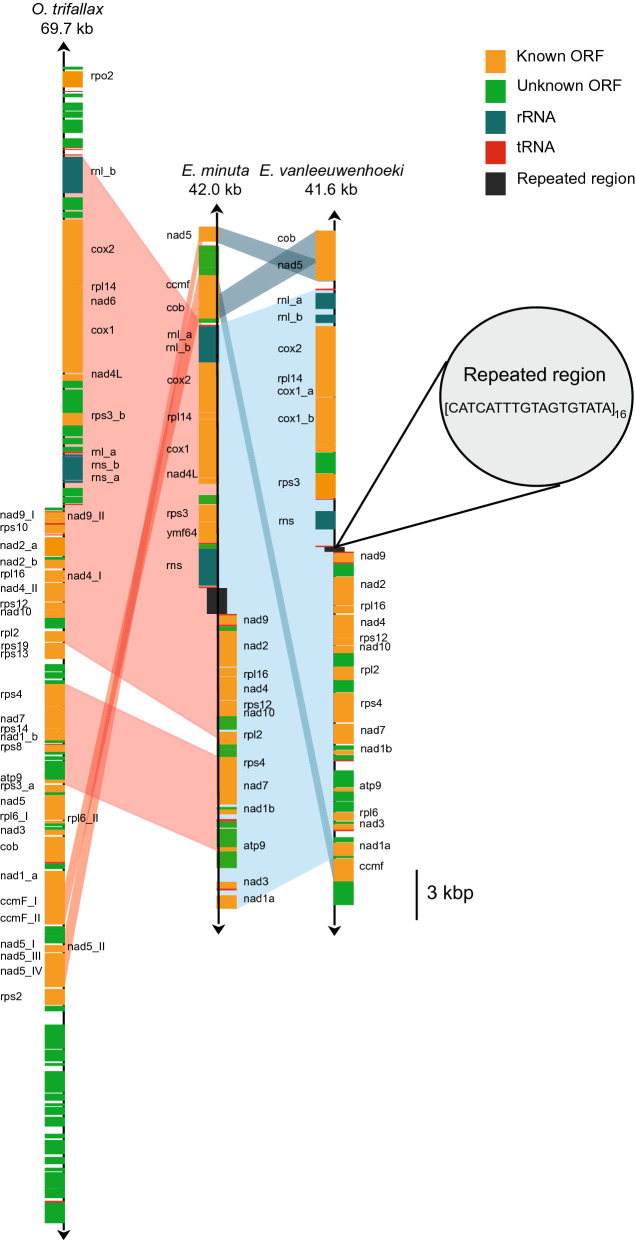
Mitochondrial gene map of *Euplotes vanleeuwenhoeki* sp. nov. The gene map of the mitochondrial genome of *E. vanleeuwenhoeki* in comparison with those belonging to *Euplotes minuta* and *Oxytricha trifallax* is represented. Homologous regions among the three genomes are indicated by pale coloured areas. Names of split genes are suffixed by a letter or a lowercase Roman numeral. The direction of transcription is indicated by an arrow at each end of the mitochondrial map.

**Figure 5:**
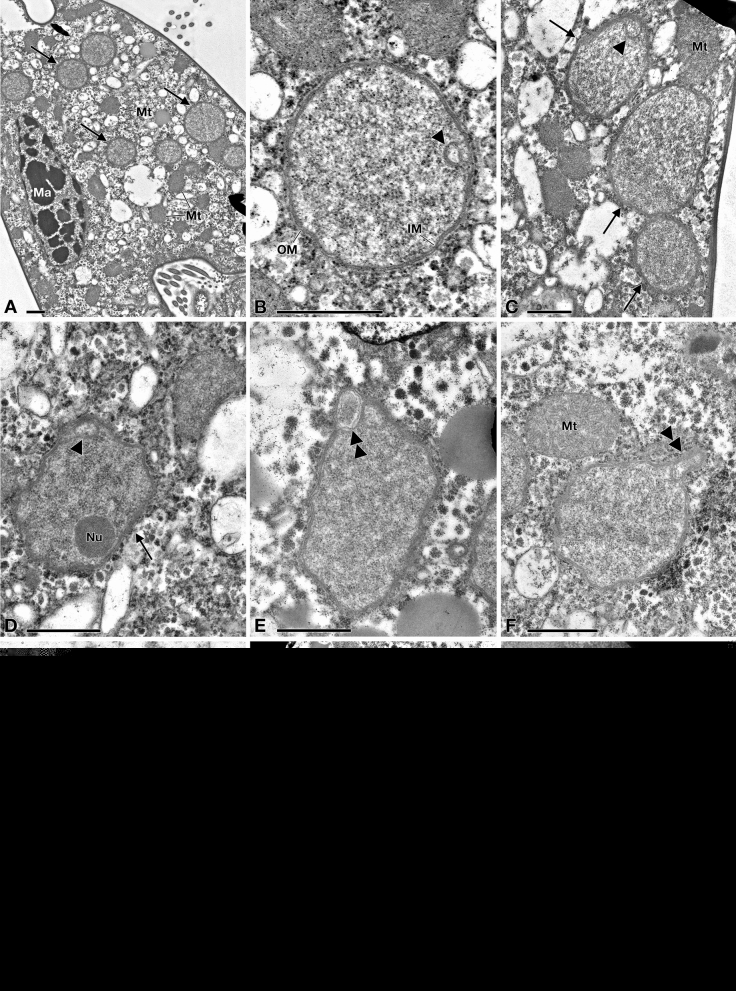
TEM pictures of "*Candidatus* Pinguicoccus supinus". (**A**) Endosymbiont cells (arrow) in host cytoplasm, lying underneath the cortex, aggregated in clusters; macronucleus (Ma) and some mitochondria (Mt), also in proximity or in apparent close contact with endosymbionts, are visible; (**B**) Closer view of “*Ca.* Pinguicoccus supinus” showing inner membrane (IM) and outer membrane (OM); an invagination of the inner membrane (IM) is present (arrowhead); (**C**) Different cell shapes of three “*Ca.* Pinguicoccus supinus” specimens (arrow), from rounded to ovoid; the IM is invaginated (arrowhead); (**D**) Endosymbiont cell with an irregular shape, with an evident nucleoid (Nu) and an invagination of the IM is present (arrowhead); (**E**,**F**) “*Ca.* Pinguicoccus supinus” cells showing evagination of the outer membrane (double arrowhead); (**F**) Endosymbiont cell in proximity to a mitochondrion; (**G**) “*Ca.* Pinguicoccus supinus” showing emphasized folding (asterisk) of membrane area; (**H**) Endosymbiont cells (arrow) appear to be in close contact with lipid droplets (L); (**I**) “*Ca.* Pinguicoccus supinus” (arrow) during binary fission; the division septum is well visible. *IM* inner membrane, *L* lipid droplet, *Ma* macronucleus, *Mt* mitochondrion, *Nu* nucleoid, *OM* outer membrane. Arrow: “*Ca.* Pinguicoccus supinus” cell; Arrowhead: evagination of the outer membrane; Asterisk: folding of membrane area; Double arrowhead: section of bacterial cell folding. Bars stand for 1 µm (**A**–**F**,**H**,**I**) and 0.5 µm (**G**).

**Table 1:** Morphometric data for *Euplotes vanleeuwenhoeki* sp. nov.

Character	MIN–MAX	*X* ± SD	CV	n
**Body length**				
Live	39.5–58.0	49.1 ± 4.7	9.7	22
SI	38.9–50.3	45.6 ± 3.1	6.7	37
**Body width**				
Live	24.9–37.8	32.7 ± 3.8	11.6	22
SI	21.6–35.5	29.1 ± 4.1	14.0	37
**Macronucleus length**				
FS	28.9–48.6	36.6 ± 4.5	12.4	15
**Macronucleus width**				
FS	4.3–7.2	5.7 ± 1.0	17.3	15
**Micronucleus diameter**				
FS	1.8–2.3	2.0 ± 0.2	9.0	15

Measures are reported in µm.

*MIN* minimum value, *MAX* maximum value, *X* arithmetic mean, *SD* standard deviation, *CV* coefficient of variation (%), *n* number of specimens analyzed, *SI* silver impregnation, *FS* Feulgen staining.

Phylum Ciliophora Doflein, 1901.

Class Spirotrichea Bütschli, 1889.

Subclass Euplotia Jankowski, 1979.

Order Euplotida Small and Lynn, 1985.

Family Euplotidae Ehrenberg, 1838.

Genus *Euplotes* Ehrenberg, 1831.

#### Diagnosis

Size in vivo (*X* ± SD) 49.1 ± 4.7 × 32.7 ± 3.8 μm. Dorso-ventrally flattened, with an oval to ellipsoidal shape. “C–shaped” or “3-shaped” macronucleus and a single micronucleus. Dargyrome of double-*eurystomus* type, 7–8 dorso-lateral ridges, with 13–14 dikinetids in the mid–dorsal row. About 22–29 adoral membranelles. Cirri pattern: ten fronto-ventral, five transverse, two marginal, and two caudal cirri. Freshwater.

#### Type locality

Freshwater emissary of Kolleru Lake, in the proximity of the Allapadu-Kolletikota road (16°36′05.0″N 81°18′47.8″E), West Godavari District of Andhra Pradesh, India. This species inhabits freshwater sites covered by *Eichhornia* sp. (water hyacinth).

#### Etymology

We dedicated this new species of *Euplotes* to Antoni Philips van Leeuwenhoek (1632–1723), Dutch optician and naturalist. Van Leeuwenhoek is best known for his pioneering work in microscopy and for his contributions toward the establishment of microbiology as a scientific discipline. For this reason, he is also known as "the father of microbiology", being one of the first microscopists and microbiologists.

#### Type material

The slide with the silver-stained holotype specimen (indicated with a black circle of ink on the coverslip) and some paratype specimens has been deposited in the collection of the “Museo di Storia Naturale dell’Università di Pisa” (Calci, Pisa, Italy) with registration number "2019-1". Two slides with silver-stained paratype specimens (indicated with a black circle of ink on the coverslip) were deposited in the collection of the Natural History Museum of London (registration number: NHMUK 2019.3.16.1), and in the collection of the Unit of Zoology-Anthropology of the Department of Biology at Pisa University (registration number: UNIPI_2019-1), respectively.

#### Morphological description

Size (*X* ± SD) in vivo 49.1 ± 4.7 × 32.7 ± 3.8 μm. Size after silver staining 45.6 ± 3.1 × 29.1 ± 4.1 μm. Cell reduction after fixation: 8%. Cells dorso-ventrally flattened, with an oval to ellipsoidal shape (Fig. 1). Right margin usually straight or slightly convex, left margin tapered in the anterior, becoming convex in the mid–body, and both ends are rounded (Fig. 1A). Ciliates can crawl on the substrate and swim freely in the medium. Cytoplasm transparent with some roundish, yellow granules; few food vacuoles containing green algae and bacteria (Fig. 1B,C). Single contractile vacuole located at the level of transverse cirri (Fig. 1B). On dorsal side, cortical ampules arranged around each bristle form conspicuous rosettes with their cortical insertions (Fig. 1C). Macronucleus (Ma) “C-shaped” or “3-shaped” (size: 36.6 ± 4.5 × 5.7 ± 1.0 μm) with irregularly dense chromatin, and a single, roundish micronucleus (Mi) (diameter: 2.0 ± 0.2 μm), usually located in a small depression close to Ma (Fig. 1D).

Dargyrome of the double–*eurystomus* type, with two rows of polygonal alveoli between each pair of dorsolateral kineties (Fig. 1E,G,I). Dorsal surface crossed by three longitudinal furrows (i.e. right marginal, median, and left marginal), reaching the posterior region of the cell (Fig. 1G). Six dorsal kineties plus 1–2 lateral, three in correspondence of dorsal furrows, carrying short bristle-like cilia (Fig. 1G); the leftmost kinety is placed in a slightly ventrolateral position (Fig. 1J). Mid–dorsal row containing up to 13–14 dikinetids, while other five dorsal rows contain 11–14 dikinetids (in detail, from the left, row1: 11; row2: 12; row3: 13–14; row5: 12–13; row6: 11–13) (Fig. 1E).

On the ventral side, invariably ten frontoventral cirri (FVC), five transverse cirri (TC), two well developed caudal cirri (CC), and two marginal cirri (MC) on the left side, in the posterior end of the cell (Fig. 1F,H,J). Argyrome is highly irregular (Fig. 1H,J) and the ventral surface presents five longitudinal ridges hosting cirral insertions; the three ridges on the left are more prominent (Fig. 1H). The first and the fifth ridges reach the posterior part of the cell at the level of the CC, while the other three ridges terminate beyond the TC (Fig. 1H).

Narrow peristome, extending for about 63% of the body length, on the ventral side. Adoral zone of membranelles (AZM) comprises 22–29 membranelles, starting at the top of the cell, travelling down along the left side and reaching the first ventral ridge, with a slight curve towards the centre of the body, at the level of transverse cirri (Fig. 1H). Length of AZM after silver staining: 43.1 ± 1.9 μm. The paroral membrane (4.7 ± 0.4 μm in length) appreciable in silver stained specimens (Fig. 1F), and in SEM-processed specimens (Fig. 1J), although carrying cilia shorter than those forming the AZM. Morphometric data are shown in Table 1.

#### Fine structure

The fine structure of *E. vanleeuwenhoeki* (Fig. 2) matches that of the other previously described *Euplotes* species, in general showing typical features^42–47^. Under the cell cortex flat alveoli are present (Fig. 2A–F,H–J). On the dorsal side, somatic cilia consisting of dikinetids (Fig. 2A,C) are deeply inserted into the cytoplasm (~ 1.4 µm); from kinetosomes only a single bristle-like cilium emerges (Fig. 1C,G). In the bristle pit, some filamentous material is sometimes visible (Fig. 2B). This is likely released by cortical ampules, the typical exocytotic organelles associated with both *Euplotes* dorsal bristle and compound ciliary organelles of the ventral surface; these organelles probably represent specialized compartments of the cell in which materials that need to be excreted are accumulated, stored, and released according to the requirements of the different *Euplotes* species^48^. Ampules associated with dorsal bristles of *E. vanleeuwenhoeki* appear elongated (size: ~ 1.6 × 0.3 µm) and usually empty possibly also due to the fixation procedure (Fig. 2C). Membranelles bordering the upper and left side of the oral cavity are separated from each other by ridges (Fig. 2D). Each membranelle of AZM consists of three rows of cilia: two equally long plus a shorter one (Fig. 2D). Axonemes contain many electron dense granules (Fig. 2D). Kinetosomes of membranelles are linked at their base. (Fig. 2E). A polystichomonad paroral membranelle is inserted on the right margin of the terminal oral cavity; its cilia appear linked to each other at the kinetosome level (Fig. 2D,E). Many flat, electron lucid pharyngeal disks are associated to the base of the cytostome, in correspondence of AZM bases (Fig. 2E,F). Macronucleus contains large pieces of chromatin and large nucleoli (Fig. 2F,G). Micronucleus consists of fine chromatin (Fig. 2G). A single contractile vacuole with an irregular silhouette is observed near a transverse cirrus (Fig. 2H). On the ventral side, kinetosomes of cilia forming cirri contain large electron dense granules (Fig. 2I). Mitochondria show variable shape and size and typical tubular cristae (Fig. 2B,C,E,F). Lipidic reserve substances consist of large granules; polysaccharidic reserve substances are represented by rosettes of glycogen abundantly and sparsely distributed throughout the cytoplasm (Fig. 2G,H). Large, irregular phagosomes are also present, with various content in different digestion stages (Fig. 2J).

Numerous, morphologically similar endosymbiotic bacteria, presenting variable shape and size, are located in the cytoplasm (Fig. 2): a detailed morphological description is presented below.

#### Gene sequence

The 18S rRNA gene sequence of *E. vanleeuwenhoeki* (strain KKR18_Esm) obtained from PCR resulted 1849 bp long, and it has been deposited in NCBI GenBank database with the accession number KY855568. The 18S rRNA gene sequence of *E. vanleeuwenhoeki* showed the highest identity with sequences of *Euplotes* cf. *antarcticus* (FJ998023) and *E. trisulcatus*, (EF690810): 99.0% (3 gaps, 16 mismatches) and 98.7% (13 gaps, 19 mismatches), respectively (Supplementary Table 1).

#### Phylogeny

The 18S rRNA gene-based phylogeny placed *E. vanleeuwenhoeki* in the so-called “clade A” of genus *Euplotes*^49, 50^, clustering together with *Euplotes* cf. *antarcticus* (FJ998023; Gao and Song unpublished) and with *E. trisulcatus*, (EF690810; Schwarz and Stoeck unpublished), with high statistical support (1.00/100%) (Fig. 3). This clade resulted sister to a clade comprising sequences attributed to *E. charon* (AF492705), *E. magnicirratus* (AJ549210), and *E. euryhalinus* (EF094968, JF903799) group (Fig. 3) (see later discussion on species attribution).

#### Mitochondrial genome

The assembly resulted in a single linear contig 41,682 bp long with a GC content of ~ 25%, representing the complete mitochondrial genome of *E. vanleeuwenhoeki*. It has been deposited in NCBI GenBank database with the accession number MK889230. It contains 36 protein coding genes and 16 tRNA genes. The genome presents the 16S rRNA and 23S rRNA genes split in two loci, with the 23S rRNA further divided into two genes, separated by a short interposing region of approximately 350 nucleotides (Fig. 4). The predicted direction of the transcription is away from a central region constituted of low-complexity repeated units (Fig. 4). The splitting of the rRNA genes and the presence of a central repeat region is a common feature shared by the two so far investigated *Euplotes* mitochondrial genomes (*Euplotes minuta* and *Euplotes crassus*)^51^ and by that of *Oxytricha trifallax*^52^. The novel genome shows an overall synteny with the mitochondrion of of *Euplotes minuta, Euplotes crassus*^51^, and *Oxytricha trifallax*^52^, with the exception of the two terminal regions, which show a different structure with respect to the other three genomes (Fig. 4).

#### Microbial consortium

To investigate the presence of possible bacteria related to *E. vanleeuwenhoeki* the whole sequenced DNA material was checked for the presence of 16S rRNA gene sequences (for detail see “Endosymbiont genome assembly and annotation” in “Experimental procedures” section). The screening of the preliminary assembly for bacterial 16S rRNA genes allowed to identify the presence of a single microorganism associated to *E. vanleeuwenhoeki*. Further analyses proved that this bacterium was localized in the cytoplasm of the ciliate, and that it was a novel endosymbiont we named “*Ca.* Pinguicoccus supinus” (see “Endosymbiont characterization” section). No other bacterial 16S rRNA gene sequence was detected in the sequencing reads. Moreover, most of the other contigs that were preliminary flagged as bacterial from the best megablast hit in the Blobology pipeline actually belonged to the mitochondrial or nuclear genome of *Euplotes*. In addition, contigs that were short (< 600 bp) or at a very low coverage (< 30×) were considered as from undetermined origin, possibly representing only minor contaminations. In both cases these contigs were discarded from the analysis (Supplementary Table 2).

### Endosymbiont characterization:* “Candidatus* Pinguicoccus supinus” gen. nov. sp. nov. (Figs. 5, 6, 7, 8, 9 and 10)

#### Morphological description

“*Ca.* Pinguicoccus supinus” gen. nov. sp. nov. is a roundish-ovoid bacterium detected in the cytoplasm of *E. vanleeuwenhoeki* (Fig. 5) with a diameter of 1.3–2.3 µm (on average (*X* ± SD): 1.9 ± 0.3 µm). It usually lies beneath the ciliate cortex, often in clusters of several individuals (Fig. 5A). Although the most common bacterial shape observed is rounded (Fig. 5B), sometimes ovoid (Fig. 5C) and irregular (Fig. 5D,E) individuals can be detected as well. This cell shape plasticity might possibly be due to the pressure exerted by the host cytoplasm on the ductile body of the bacterium. No symbiosome is observed to isolate the endosymbiont from ciliate cytoplasm (Fig. 5). “*Ca.* Pinguicoccus supinus” is delimited by a double membrane with a thin space between the two layers possibly corresponding to the paryphoplasm (Fig. 5B), the intracellular space defined for the first time by Linsday and colleagues^53^. In several individuals, the increase of membrane area is visible: in some cases, a slight invagination of the inner membrane occurs (Fig. 5B–D), while in others the evagination of the external membrane can be observed (Fig. 5E,F). In the latter case, different inclusions of unknown origin have also been observed in the space between the inner and outer membranes (Fig. 5E). The bacterial cytoplasm (possibly corresponding to the pirellulosome^53^) generally appears homogeneous and a compact, more electrondense region, likely corresponding to bacterial nucleoid, is visible in some bacteria with an eccentric localization (Fig. 5D,E,H,I). Occasionally, specimens show a very emphasized folding of the membrane area, making it difficult to recognize whether the folding comes from the inner or the outer membrane (Fig. 5G). Out of a total of ~ 80 endosymbionts observed in the thin section, roughly one third are in proximity (i.e. at a distance of ~ 0.25 µm or less) of mitochondria (Fig. 5A–C,F,I) and one fifth are in proximity to lipid droplets (Fig. 5E,G,H). Intriguingly, in these cases, bacterial double membrane is even seen somehow in direct contact with mitochondrial external membrane (Fig. 5C) and lipid droplets (Fig. 5H). *“Ca.* Pinguicoccus supinus” reproduces in the host cytoplasm by binary fission and has, apparently, a typical symmetrical division (Fig. 5I).

**Figure 6:**
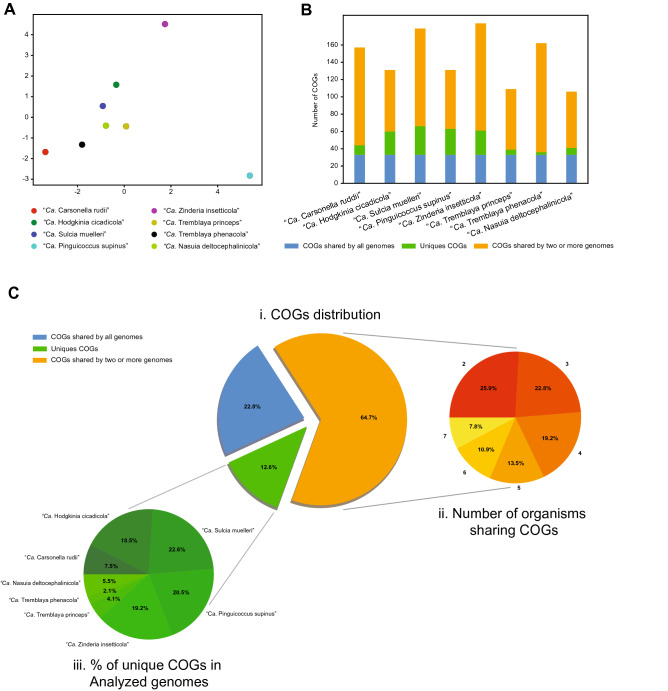
COG analysis of “*Candidatus* Pinguicoccus supinus” and other bacteria with highly reduced genome. (**A**) Principal Component Analysis of numerosity in COG classes; explained variance by Component one 32%; explained variance by Component two 24%; (**B**) Distribution of COGs in each analysed genome; (**C**) Distribution of COGs, showing: i. percentage of COGs shared by all the analysed genomes, COGs unique for each genome, and COGs shared by at least two genomes; ii. Number of organisms sharing COGs. Each set groups together the COGs shared by a given number of organisms (i.e. 1, 2, 3…7), regardless of their identity; iii. Percentage of unique COGs in analyzed genomes.

**Figure 7:**
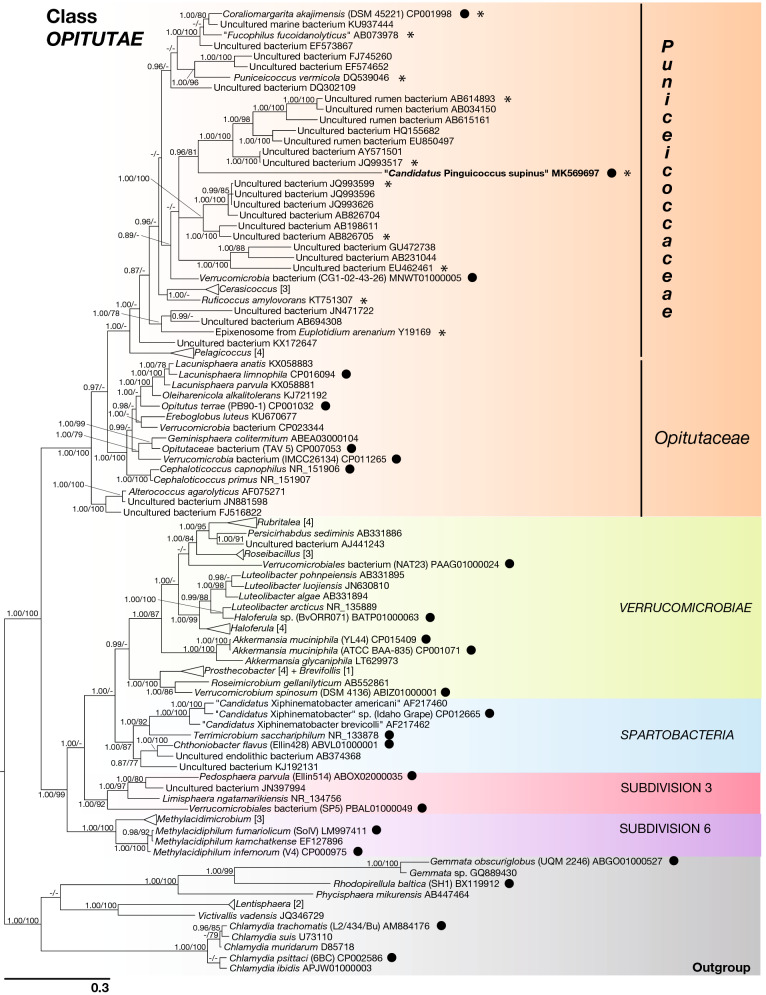
Phylogenetic tree of Phylum *Verrucomicrobia*, based on the 16S rRNA gene. The phylogenetic position of "*Candidatus* Pinguicoccus supinus" is shown. Numbers associated to nodes represent posterior probability and bootstrap values, respectively (only values above 0.80–75 are shown). Black circles indicate organisms also employed in phylogenomic analysis (Fig. 8). Asterisks indicate sequences employed in the 16S rRNA gene screening on IMNGS (Fig. 10; Supplementary Table 6). Numbers in square brackets, associated to collapsed branches, indicate how many sequences are not shown (for list of hidden sequences see Supplementary Table 9). Sequence obtained in the present work is in *bold*.

**Figure 8:**
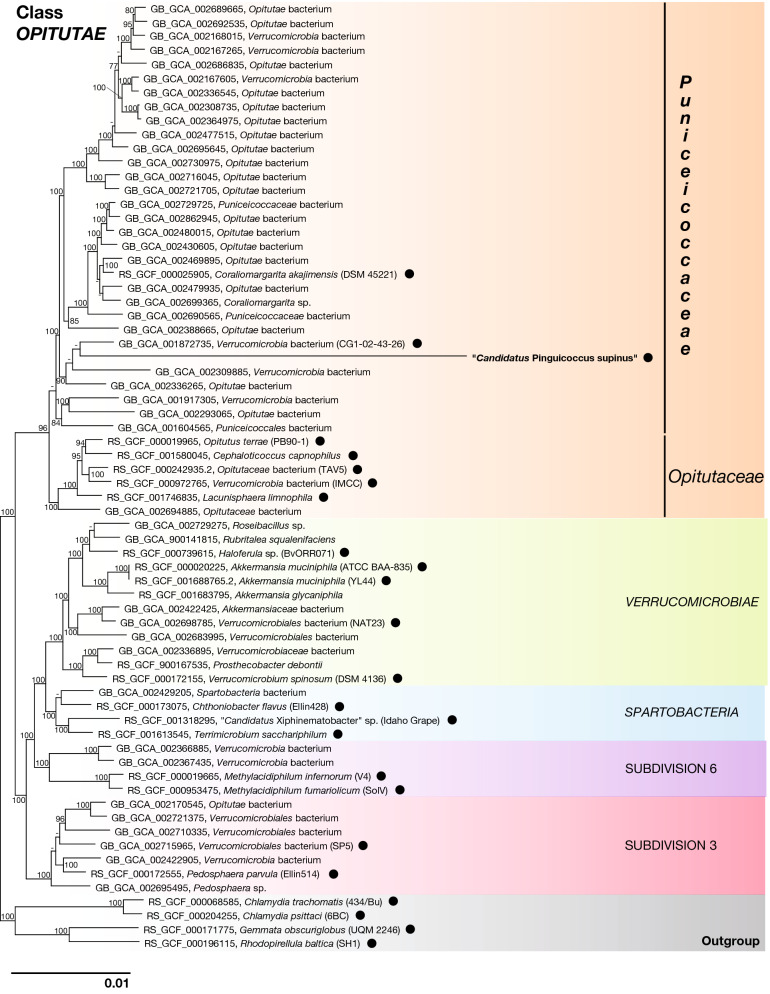
Phylogenomic tree of *Verrucomicrobia*, showing evolutionary relationships of "*Candidatus* Pinguicoccus supinus". Numbers associated to nodes represent bootstrap values (only values above 75 are shown). Black circles indicate organisms also employed in phylogenetic analysis (Fig. 7). Genome of “*Ca.* Pinguicoccus supinus” obtained in the present work is in *bold* (accession number: CP039370).

**Figure 9:**
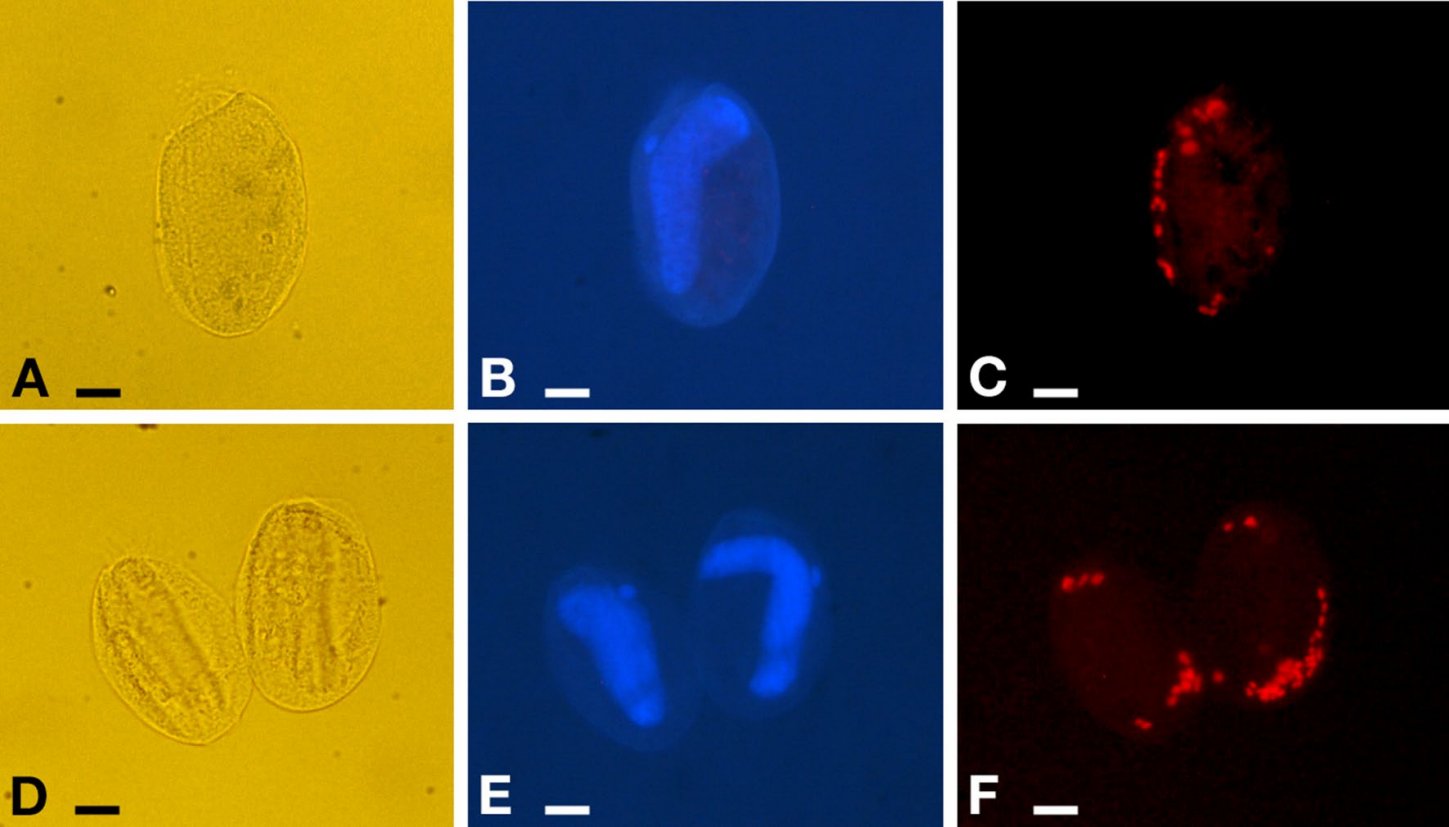
Fluorescence in situ hybridization experiments on *Euplotes vanleeuwenhoeki* sp. nov. (**A**–**C**) Specimen hybridized with probe EUB338 VII. (**D**–**F**) Two specimens hybridized with probe Pingui_1174. (**A**,**D**) Pictures at DIC microscope of fixed cells; (**B**,**E**) DAPI staining, showing position of nuclear apparatus; (**C**) Cell positive to probe EUB338 VII, fluorophore emitting in red (Cyanine-3); (**F**) Cell positive to probe Pingui_1174, fluorophore emitting in red (Cy-3). Bars stand for 10 µm.

**Figure 10:**
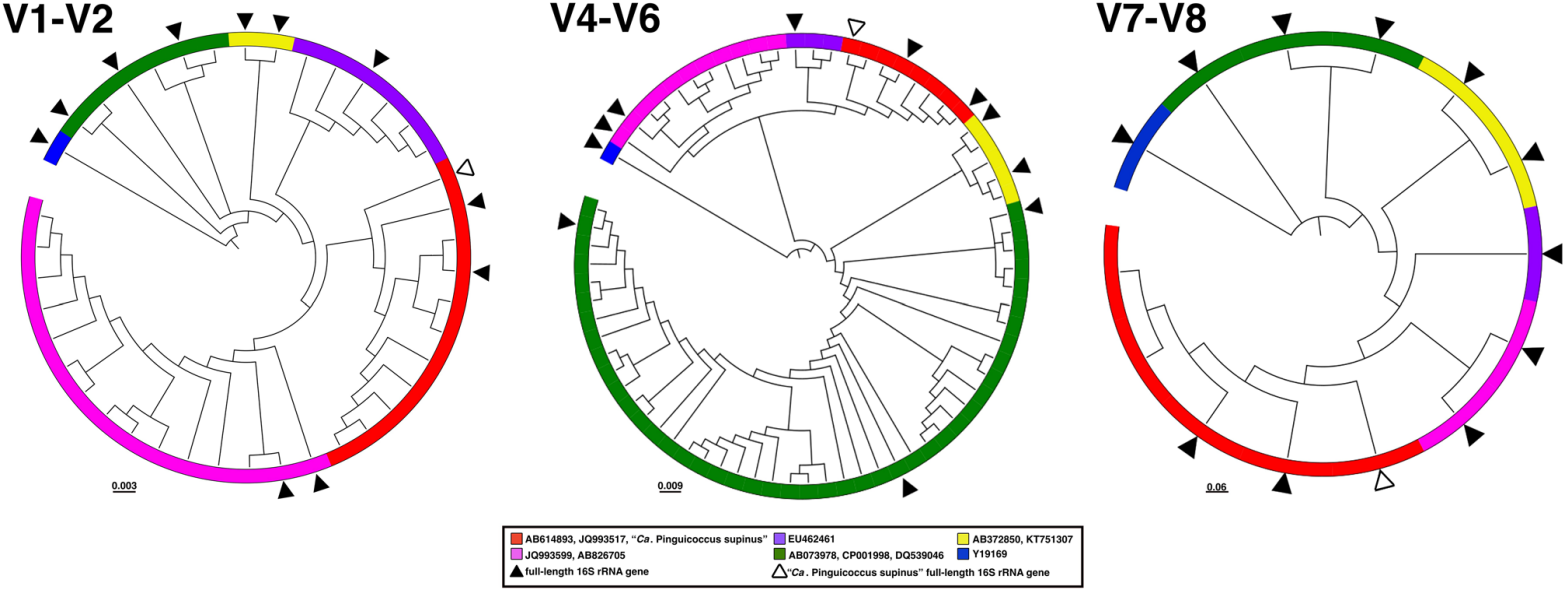
Diversity of "*Candidatus* Pinguicoccus supinus" based on 16S rRNA gene amplicon search in IMNGS. 16S rRNA gene hypervariable regions phylogenetic trees. OTUs were clustered with 99% identity and were longer than 300 bp. A total number of 90 OTUs were separated for each hypervariable region taken into analysis: 29 for V1–V2, 60 for V4–V6, 2 for V7–V8. Complete 16S rRNA gene was employed in the analysis to enlighten the diversity of “*Ca*. Pinguicoccus supinus”. White triangle indicates the “*Ca*. Pinguicoccus supinus” full-length 16S rRNA gene; black triangle indicates the full-length 16S rRNA gene.

#### Gene sequence

The 16S rRNA gene sequence of “*Ca.* Pinguicoccus supinus” resulted 1517 bp long and has been deposited in NCBI GenBank database with the accession number MK569697. Best BLAST hits of the obtained 16S rRNA gene against NCBI database were sequences from uncultured bacteria: JQ993517 and MNWT01000005, AB826705, and AY571501 (identity values: 78.8%, 78.3%, and 78.2%, respectively). The best hit with a cultured bacterium was 76.8% with *Ruficoccus amylovorans* (KT751307) (*Verrucomicrobia*, *Opitutae*, *Puniceicoccaceae*). In general, identity values with the closest relatives resulted quite low (75.7–78.8%) (Supplementary Table 3).

#### Genome assembly

The assembly of the symbiont’s genome resulted in a single circular chromosome, 163,218 bp long with a GC content of 25.1%. The complete genome sequence of “*Ca.* Pinguicoccus supinus” has been deposited in NCBI GenBank database with the accession number CP039370. It contains 168 protein coding sequences, 34 tRNA genes and a single rRNA operon composed of a 16S rRNA gene, a 23S rRNA gene and a 5S rRNA gene. The overall coding percentage is 92.3%. The ORFs were subjected to clusters of orthologous groups (COGs) classification, and 131 COGs were identified, most of which were related to the general cellular function categories: J (translation, ribosomal structure, and biogenesis), O (post-translational modification, protein turnover, chaperons), M (cell wall/membrane/envelope biogenesis), and I (lipid transport and metabolism), for a total of 112 COGs (Supplementary Table 4). This repertoire is a subset of the previously characterized *Verrucomicrobia*, i.e. without any exclusive metabolic pathway or gene (Supplementary Table 5). Considering the drastic genome reduction of “*Ca.* Pinguicoccus supinus”, we decided to perform comparative analyses using as reference the few other symbiotic bacteria with highly reduced genome although belonging to unrelated lineages (i.e. *Bacteroidetes, Alphaproteobacteria, Gammaproteobacteria, Betaproteobacteria*).

A Principal Component Analysis (PCA) based on retrieved COGs was able to capture almost 56% of the whole variance (Fig. 6A) in the COG dataset, and thus capturing the functional diversity among the analyzed genomes. While almost all the bacteria with highly reduced genome cluster together (Fig. 6A), the variance explained by the first component positions “*Ca.* Pinguicoccus supinus” is remarkably distant. This component is mainly correlated with the COG classes Q—Secondary metabolite biosynthesis, transport and catabolism (eigenvalue: 0.36), I—Lipid transport and metabolism (0.34), and K—Transcription (0.33). The variance explained by the second component separates “*Ca.* Zinderia insecticola” from the other bacteria with highly reduced genome. This component is mainly correlated with COG classes P—Inorganic ion transport and metabolism (0.42), H—Coenzyme transport and metabolism (0.39), and C—Energy production and conversion (0.33) classes.

COG analysis also showed the presence of a set of 33 genes shared by all the small genome bacteria in analysis (Fig. 6B,C), mostly related to DNA replication, transcription, and translation. Although the metabolic capability of “*Ca.* Pinguicoccus supinus” is, in general, similar to that of other highly reduced genomes^40,54^ (88% of COGs shared with at least another analysed genome; Fig. 6B,C), the novel genome does not code for the synthesis of any amino acid. Moreover, no catalytic subunit of the DNA polymerase was identified. “*Ca.* Pinguicoccus supinus” possesses 30 genes (12% of the total retrieved COGs in this bacterial genome) that are absent in all the other tiny genomes, mostly related to COG classes I (lipid transport and metabolism) and M (cell wall/membrane/envelope biogenesis). In general, with respect to the other analyzed bacteria, this endosymbiont includes a richer set of genes involved in fatty acid biosynthesis, in glycosylation and glycan modification (Supplementary Table 4).

#### Phylogeny and phylogenomics

The 16S rRNA gene-based phylogeny showed “*Ca.* Pinguicoccus supinus” as a member of the family *Puniceicoccaceae* (*Verrucomicrobia, Opitutae*) (Fig. 7). It clustered with sequences from uncultured organisms, forming a clade related to the genera *Coraliomargarita, “Fucophilus”, Cerasicoccus,* and *Ruficoccus* (Fig. 7). The long branch of “*Ca.* Pinguicoccus supinus” suggests a higher evolutionary rate with respect to related *Verrucomicrobia*. Moreover, we observed that the inclusion of the “*Ca.* Pinguicoccus supinus” sequence in the *Verrucomicrobia* tree reduces the values of statistical supports for the nodes of the *Puniceicoccaceae* clade (data not shown). This is consistent with the occurrence of a long branch attraction phenomenon between “*Ca.* Pinguicoccus supinus” and the outgroup, which destabilizes *Puniceicoccaceae* despite high taxon sampling^55^.

The result of the phylogenomic analysis is fully consistent with the phylogeny of the 16S rRNA gene, confirming the position of the endosymbiont inside the phylum *Verrucomicrobia* and in the class *Opitutae* (Fig. 8). “*Ca.* Pinguicoccus supinus” clustered together with uncultured organisms (GB_GCA_001872735, GB_GCA_002309885, GB_GCA_002336265), in a clade related to *Coraliomargarita akajimensis* (RS_GCF_000025905), and thus inside the family *Puniceicoccaceae* (Fig. 8).

#### Localization inside host cell

FISH experiments showed 100% of *E. vanleeuwenhoeki* cells positive to specifically designed probes for “*Ca.* Pinguicoccus supinus” (Fig. 9). The number of endosymbionts per host cell ranged from 12 to 36 bacteria (*X* ± SD: 25.1 ± 7.3; n = 16). The symbionts showed a peculiar pattern of distribution in almost all the observed *Euplotes* cells: beneath the cortex of the host, in clusters of several bacteria grouped at one side of the cell (Fig. 9C), or more commonly at the two poles (Fig. 9F). These observations support data from TEM analysis, showing “*Ca.* Pinguicoccus supinus” generally lying close to the cortex and forming clusters with other conspecifics.

#### Diversity and environmental screening of the endosymbiont

The Integrated Microbial Next Generation Sequencing (IMNGS) platform^56^, was used to investigate the molecular diversity and environmental distribution of sequences showing high identity with the novel endosymbiont of *Euplotes*. The 16S rRNA gene of the endosymbiont was queried at 95% similarity threshold, and a total number of 4572 sequences were obtained and subsequently used to investigate the molecular diversity of 16S rRNA gene hypervariable regions. Sequences were clustered in 90 OTUs with 99% threshold identity, then OTUs were grouped according to their hypervariable region in V1–V2, V4–V6 and V7–V8, and phylogenetic trees were inferred accordingly (Fig. 10). Phylogenetic trees showed that six diverse groups can be distinguished for all hypervariable regions considered, and their phylogenetic relations are in agreement with those shown by the full-length 16S rRNA gene phylogenetic reconstruction (Fig. 10). The first group (red) includes the novel endosymbiont and related sequences (AB614893, JQ993517; Fig. 7), the second one (violet) is formed by sequences from uncultured bacteria (JQ993599, AB826705), the third one (purple) consists of a single uncultured bacterium (EU462461), the fourth one (green) includes *Coraliomargarita akajimensis* (CP001998), “Fucophilus fucodainolyticus” (AB073978), and *Puniceicoccus vermicola* (DQ539046), the fifth one (yellow) has *Cerasicoccus frondis* (AB372850) and *Ruficoccus amylovorans* (KT751307), and the last one (blue) includes just epixenosomes from *Euplotidium arenarium* (Y19169) (Fig. 10).

The IMNGS environmental screening also showed that metagenomic samples positive to “*Ca*. Pinguicoccus” were very limited in number, namely 0.01% of total samples. Indeed, the related sequences were present in just 32 out of a total of 303,362 samples (Supplementary Table 6). The environmental origin of positive samples is reported in Supplementary Table 6. Positive hits originated from very diverse samples, such as seawater, wastewater, microbial mats, but also potential host organisms, such as shrimps and plants (Supplementary Table 6). However, the overall abundance of positive hits was extremely low and always below 1% within each sample. The highest abundance was found in samples originating from a study on wastewater^57^, where “*Ca*. Pinguicoccus” abundance ranged between 0.005 and 0.752% (Supplementary Table 6).

## Discussion

### First biont: *Euplotes vanleeuwenhoeki*

#### Comparison with congeners

*Euplotes vanleeuwenhoeki* showed morphological and molecular affinity with other members of the genus, such as *E. antarcticus*^58,59^*, E. trisulcatus*^60^*, E. euryhalinus*^61^*, E. charon*^62,63^*,* and *E. magnicirratus*^60^. Indeed, they shared the same dargyrome and frontoventral cirri patterns (for details see Table 2), and clustered together in our molecular phylogeny (Fig. 3). As for its morphology, *E. vanleeuwenhoeki* was particularly similar to *E. trisulcatus*^60^. Nevertheless, the combination of several characters (i.e. posterior end rounded *versus* pointed; peristome length, number of membranelles in the AZM, number of dikinetids in mid-dorsal row—for details see Table 2), the overall body shape, the type of habitat (freshwater *vs* marine), and the 18S rRNA gene sequence supported its attribution to a novel species.

**Table 2:** Morphological comparison between *Euplotes vanleeuwenhoeki* sp. nov. and selected congeners.

Character	*Euplotes vanleeuwenhoeki*	*E. trisulcatus*	*E. antarcticus*	*E. euryhalinus*	*E. charon*	*E. magnicirratus*
Body size in vivo (µm)	40–58 × 25–38	35–50 × 25–40	90–145 × 30–80	50–62 × 26–38	70–100 × 65–90	51–65 × 36–44
Body shape	Elongated ellipsoidal; posterior end rounded	Elongated ellipsoidal; posterior end pointed	Elongated ellipsoidal; posterior end rounded	Elongated oval; posterior end pointed	Oval; posterior end rounded	Oval; posterior end rounded
Peristome (% of the body length)	63	75	65	67	75	75
Number, type of dorsal structures	3, prominent furrows	3, prominent furrows	7–10, prominent DR	Several, inconspicious DR	7, prominent DR	Several, prominent DR
Number of membranelles in AZM	22–29	25–36	46–56	26–28	51–60	49–52
Dargyrome type	Double-*eurystomus*	Double-*eurystomus*	Double-*eurystomus*	Double-*eurystomus*	Double-*eurystomus*	Double-*eurystomus*
Number of dorsolateral kineties	7–8	7	10–11	10	9–10	8
Number of dikinetids in mid-dorsal row	13–14	7–10	11–21	10	22	13–17
Number of FVC	10	10	10	10	10	10
Number of TC	5	5	5	5	5	5
Number of CC	2	2	3–4	2–3	2–4	2
Number of MC	2	2	2	2	2	2
Habitat	Freshwater	Marine	Marine	Euryhaline	Marine	Marine
References	This study	^60^	^59^	^61^	^62,63^	^60^

*AZM* adoral zone of membranelles, *CC* caudal cirri, *DR* dorsal ridge, *FVC* frontoventral cirri, *MC* marginal cirri, *TC* transverse cirri.

Unfortunately, the sequences closest to that of *E. vanleeuwenhoeki* in our phylogeny reconstruction (i.e. *E. magnicirratus*—AJ549210^64^; *E. charon*—AF492705^65^; *E. euryhalinus*—EF094968^66^; JF903799 (Keerthi and Reddy unpublished); *E. trisulcatus* EF690810 (Schwarz and Stoeck unpublished), *Euplotes* cf. *antarcticus*—FJ998023 (Gao and Song unpublished) are not accompanied by any morphological description. Consequently, data used in our morphometric comparison inevitably derive from previous, exclusively morphological studies^59–63^. Moreover, there is no certainty that the above-mentioned *Euplotes*-derived sequences were properly attributed to the correct ciliate species; thus, a certain cautiousness is recommended until a comprehensive redescription of such organisms, joining morphology and molecular data, has been performed. In this somehow unclear landscape, for the sake of completeness, we decided to include also *E. charon* in our comparison among different *Euplotes* species (Table 2)*,* because it shares some basic features with *E. vanleeuwenhoeki,* (i.e. dargyrome, and frontoventral cirri pattern), although the phylogenetic position of this species is far from being resolved^67^. Indeed, the sequence AF492705, under the name of *E. charon*, is not linked to any morphological description^65^; this sequence was used by Shao and colleagues^63^ as a molecular reference in their redescription of *E. charon*, for which, unfortunately, they used a different *Euplotes* strain for morphological analysis. However, there are several other available sequences that are named after *E. charon,* but none are supported by any morphological data [i.e. AJ305249^64^; JF694043^68^; FJ87077-80 (Huang and Song unpublished)] and these sequences cluster far away from *E. vanleeuwenhoeki.* A critical revision of the species *E. charon* using at least integrative taxonomy is consequently highly recommended.

As a general remark, *E. vanleeuwenhoeki* shares with many other species the plesiomorphic condition of the genus, i.e. the occurrence of ten FVC and a double dargyrome of *eurystomus*-type (e.g.: *E. alatus, E. antarcticus, E. balteatus, E. charon, E. crenosus, E. curdsi, E. enigma, E. euryhalinus, E. focardii, E. harpa, E. inkystans, E. magnicirratus, E. neapolitanus, E. octocirratus, E. palustris, E. parabalteatus, E. platysoma, E. plicatum, E. polycarinatus, E. qatarensis, E. quinquecarinatus, E. rariseta, E. trisulcatus*, and *E. tuyraui*). Although these two features have always been reported in morphological description, due to their plesiomorphic status they are of limited use for taxonomic identification^67^. As an example, some authors^69^ even proposed to synonymise almost all species possessing ten FVC and a double dargyrome under the name of *E. charon*. Obviously, nowadays, this proposal is not acceptable on the basis of molecular data^50,67,70^ and because of other morphological features characterizing some of the organisms (e.g. dorsal furrows/ridges, and body shape and proportions).

It has been proved that, in many cases, this ancestral condition underwent severe, independent modifications during the evolutionary history of the genus^49,64,67^. Indeed, the double dargyrome evolved in other different patterns, such as “single”, “double *patella*-type”, “multiple”^60^, and “complex”, in many distinctive events^49,67^. The dargyrome modification was explained either by increasing in number or by unification of alveoli^49,67,71^. It is not by chance that some *Euplotes* species present a great phenotypic plasticity, determined by particular physiological and/or ecological conditions, such as the presence of a predator and/or food availability (i.e. *E. focardii*^72^, *E. balteatus*^73^, *E. octocarinatus*^74,75^, and *E. variabilis*^76^. Similarly, one or more FVC were independently lost many times, as also evidenced by the vestiges still detectable in some *Euplotes* species^49,64,67,77,78^.

#### Mitochondrial genome

Ciliates possess very peculiar mitochondrial genomes, being among the first to be identified as linear^79,80^ and showing several split rRNA genes^81^ and protein genes^82^. To our best knowledge, in ciliates, the set of potentially split genes in the mitochondrial genome includes *nad1, nad2, rps3, rnl* and *rns*. This list now includes the *cox1* gene, which is split in *E. vanleeuwenhoeki*. Considering that the splits in *nad1, nad2, rps3, rnl* and *rns* genes occur approximately at the same positions in the mitochondrial genomes of Spirotrichea, it has been hypothesized they could be present in the last common ancestor of *Euplotes* and *Oxytricha*^52^. However, since the *cox1* gene is split only in the *E. vanleeuwenhoeki* genome, it is likely that this represents a more recent event. The occurrence of multiple and evolutionary independent split genes in ciliates could be considered indicative of a possible inherently higher tolerance to split events in mitochondrial genes in these organisms.

The mitochondrial genome of *E. vanleeuwenhoeki* shows a high level of colinearity with the other two available *Euplotes* mitochondrial genomes. In addition, all *Euplotes* share a good colinearity with *O. trifallax* (two single-gene inversions and one single-gene transposition). Since the amino acid substitution rates in ciliate mitochondrial genomes appear to be high^52^, this result enforces the idea that mitochondrial genome colinearity can be employed in phylogenetic analysis as a valid supporting feature to define the systematics of ciliates, or to distinguish different taxonomic levels among close related species using the non-colinear terminal regions^83,84^. Indeed, mitochondrial genes, such as *cox1*, *cox2* and *rns*, are being increasingly used in taxonomy, both for phylogenetic analysis^85^, and for discriminating among species with similar morphological features. For example, mtDNA genes have been employed to reveal several cryptic species in Ciliophora^86,87^ and metazoan such as annelids^88^ and insects^89^, or to discriminate between subspecies of the bee *Apis mellifera*^90^. The limited number of available mitochondrial genome sequences of ciliates currently does not permit phylogenomic analyses based either on sequences or on colinearity. Nevertheless, we predict that such analyses will be extremely helpful, in the future, to resolve phylogenetic and taxonomic issues as already occurred in other taxa (e.g. metazoa) in which more sequences are already available^91,92^. We expect the same for ciliates as soon as enough novel mitochondrial genome sequences are available.

#### Symbiosis with a *Verrucomicrobia* member

Members of genus *Euplotes* are known to host symbiotic eukaryotes, mutualistic such as endocellular algae^46,93^ or opportunistic such as microsporidia^94^ and trypanosomes^27^. Moreover, *Euplotes* species display a certain propension to harbor one or even multiple bacterial symbionts^95–99^. This second condition appears more common if the ciliate presents the obligate symbiosis with *Polynucleobacter necessarius* or “*Ca.* Protistibacter heckmannii”^99–106^. In those cases, the secondary or tertiary symbionts, if present, frequently belong to *Alphaproteobacteria,* e.g. *Rickettsiaceae*^99,103,105,107^ or “*Ca*. Midichloriaceae”^99,102,104,108^. Also, *Gammaproteobacteria* were retrieved in different *Euplotes* species^99, 101,106,109,110^. For a detailed and comprehensive review on *Euplotes* endosymbionts see Ref.^99^.

To date, “*Ca*. Pinguicoccus supinus” as endosymbiont of *E. vanleeuwenhoeki* constitutes a *unicum*, being the first member of *Verrucomicrobia* with a highly reduced genome, and also the first found as an endosymbiont of a protist. Indeed, bacteria of the phylum *Verrucomicrobia* are mostly free-living organisms, nearly ubiquitous, present both in the soil and in wet environments^111,112^, and often retrieved in extreme habitats^113,114^. Little is known about *Verrucomicrobia* living as obligate symbionts, which at present were described in nematodes^115^, echinoderms^116^, squids^117^, and in the gut of different metazoans^118,119^, including humans^120^. The only other case of association of *Verrucomicrobia* with protists is the symbiosis between the ciliate *Euplotidium* and the so-called epixenosomes^33,121,122^ which are verrucomicrobial ectosymbionts able to extrude and defend their host against predators^6^.

### Second biont: *“Candidatus* Pinguicoccus supinus”

#### Endosymbiont morphology

The morphology of some PVC members is renowned to be peculiar. Indeed, some of them present a certain degree of cellular compartmentalization, even if some aspects of the subject are still under debate^53,123,124^. Cell compartmentalization has been proposed for some members of *Verrucomicrobia*, such as *Prostechobacter dejongeii* (*Verrucomicrobiaceae*) and *Coraliomargarita akajimensis* (*Puniceicoccaceae*) based on molecular and ultrastructural studies^125,126^. In those two bacteria, the presence of two different cellular compartments, delimited by an intracytoplasmic membrane, has been described. These two compartments were recognizable as a ribosome-free region, the paryphoplasma, and a region containing ribosomes and nucleoid, the pirellulosome. In this framework, some morphological traits of “*Ca.* Pinguicoccus supinus”, such as membrane invaginations and folding, could be considered homologous to those observed in other *Verrucomicrobia* and PVC members in general, although our data, based solely on TEM observation, definitely do not provide clear evidence of cell compartmentalization. A possible future development could be the use of cryo-electron tomography^127^.

#### Endosymbiont genome

The genome of “*Ca*. Pinguicoccus supinus” was found to be extremely small (163,218 Kbp), being, to our best knowledge, the fourth smallest bacterial genome sequenced to date (smallest being “*Ca.* Nasuia deltocephalinicola”: 112,031 bp)^128^. The novel genome is highly reduced and lacks many genes, especially compared with the relative free living *Verrucomicrobia* (range 2.2–7.3 Mbp)^114,129^ but also with other *Verrucomicrobia* symbionts, such as the ones belonging to the genus “*Ca.* Xiphinematobacter” (~ 916 Kbp)^130^. The tiniest genomes (< 500 Kb) are found in putatively ancient mutualistic symbioses, where symbionts are beneficial to their hosts’ metabolism and hosts have in turn evolved to support and control the symbiosis^40,54,128,131^. Such levels of co-evolution often also imply phenomena of severe genome reduction, with gene loss and often a subsequent modification of the nucleotide base composition^54^. This framework gives us some clues for understanding the peculiar characteristic of the novel symbiont “*Ca*. Pinguicoccus supinus”. All other symbionts with highly reduced genomes provide their hosts with nutrients that are absent in their diet. In those cases, the nutritional support role is clear, due to the fact that they retain some metabolic pathways for the biosynthesis of amino acids essential for their hosts^132^ (for a review see Ref.^54^). Moreover, several of these symbionts are co-obligate with other bacteria complementing each other for the enzymatic repertoire necessary to provide nutritional support to the host. This seems not to be the case of “*Ca*. Pinguicoccus supinus”, which is devoid of genes related to amino acids, co-factors or vitamin biosynthesis, and is the only symbiont retrieved in the *E. vanleeuwenhoeki* holobiont. The extensive gene loss and genome modification suggest that this symbiosis may have an ancient origin, and that it may play an important role for the host, although its function is yet to be understood. This case could be partly similar to that of the well-ascertained mutualist bacterium, *Polynucleobacter necessarius* in other *Euplotes* spp.^133,134^. Indeed, this bacterium is necessary for the survival of its hosts, but no precise clues on the metabolic nature of the interaction have been provided by genomic analyses^133,134^. This has been linked to the peculiar ecology of ciliates, which normally feed on free-living bacteria, and are thus unlikely to require dietary compensations from bacterial endosymbionts. It has been hypothesized that they may require help in catabolism or other undefined functions, and such a contribution would not be simple to detect by analyses of the symbionts’ genomes only^133^.

The comparison between “*Ca.* Pinguicoccus supinus” and the other symbionts with highly reduced genomes shows the presence of a core set of 33 genes retained by all these evolutionary unrelated bacteria. Those genes are mostly involved in DNA replication, transcription, and translation, as well as in protein folding and stability. Clearly, these core cellular functions are required even in bacteria with such extremely reduced genomes. Interestingly, in “*Ca.* Pinguicoccus supinus”, it was not possible to find a gene homologous to a DNA polymerase II catalytic subunit (*dnaE*), or, in general, to any other protein with predicted DNA polymerase catalytic activity. This is a striking difference with respect to the other analysed symbionts, and, to our best knowledge, it is a unique case among all bacteria. A Blast analysis on the entire preliminary assembly of the whole holobiont did not reveal the presence of any *dnaE* gene, suggesting that there is no such gene integrated in the host genome. This finding strongly suggests that “*Ca.* Pinguicoccus supinus” relies on proteins obtained from the host to replicate its own genome, analogously to what has been proposed for other essential cellular functions for other symbionts with highly reduced genomes^54^.

A common feature of the previously characterized symbiotic bacteria with highly reduced genomes is the complete lack of genes related to the production of components of the cellular envelope such as phospholipids, lipopolysaccharide, peptidoglycan and related membrane proteins^54^. Unique in this regard, “*Ca.* Pinguicoccus supinus” presents the pathway for the initiation and elongation of fatty acids, and several glycosyl transferases, although the phospholipid synthesis pathway is missing. Obligate small genome symbionts of insects are found in specialized cells, the bacteriocytes and some symbionts of unicellular eukaryotes are located in a specific cellular structure, the symbiosome, while “*Ca*. Pinguicoccus supinus” resides free in the host cytoplasm. This could imply the requirement for a more complex regulation of the membrane structure (e.g. variation of fatty acid length in phospholipids) to face a “less stable” environment, thus possibly explaining the unique genes found in the genome of “*Ca*. Pinguicoccus supinus”. Interestingly, “*Ca.* Pinguicoccus supinus” has often been observed in close contact with the host’s lipid droplets. It is not yet elucidated whether the interaction with the host’s lipids could be another reason for the retention of genes devoted to fatty acid biosynthesis.

The genome of “*Ca.* Pinguicoccus supinus” presents a non-standard genetic code (NCBI genetic code “4”), in which the canonical stop triplet UGA is recoded for tryptophan, consistently with the absence of release factor 2, implied in the normal recognition of UGA as stop. This is consistent with other highly reduced genomes^135^ and has been tentatively linked to a directional mutation pressure related to the AT/GC composition^136^, or directly linked to genome reduction and energetic constrains^135^.

#### Endosymbiont phylogeny

Both the phylogenetic and phylogenomic analyses, the first including more taxa and the second more genes, support the position of “*Ca.* Pinguicoccus supinus” in the family *Puniceicoccaceae* (*Opitutae*, *Verrucomicrobia*). We suggest that the long branch of the novel species is due to an overall high evolutionary rate, and consequently may decrease support for some nodes inside the family-clade.

It is worth noticing that our phylogenetic and phylogenomic analyses are among the most taxonomically exhaustive up to date for *Verrucomicrobia* and resulted coherent with those from previous studies^137–140^. They also highlight the necessity for systematic revision for some species and taxa (i.e. *Brevifollis gellanilyticus* clustering within genus *Prostechobacter*; Subdivision 3 and Subdivision 6 still waiting for a proper naming).

#### Endosymbiont diversity and environmental distribution

In order to evaluate how widespread and abundant the novel taxon is, we evaluated the presence of sequences related to 16S rRNA gene of *E. vanleeuwenhoeki* endosymbiont in IMNGS, which resulted to be very limited. The main possible reason of the scarcity of “*Ca*. Pinguicoccus supinus” and related sequences in online repositories resides in the fact that 16S rRNA gene primers employed in metabarcoding and metagenomic studies do not often detect certain bacterial groups, such as *Verrucomicrobia*^141,142^. Indeed, the choice of primers used in such NGS analysis should be carefully pondered as some bacterial taxa might be undetected, thus not depicting the real diversity in the environment. Our analysis shows that the 16S rRNA sequences related to the endosymbiont were present in just 0.01% of total samples, and never reached 1% abundance in positive samples, a number of which resulted to be microbiome studies of multicellular organisms (Supplementary Table 6). The hypervariable region diversity analysis confirmed the limited presence of sequences from these bacteria sequences in metabarcoding samples. Extracting from the databases sequences similar to “*Ca.* Pinguicoccus supinus” and performing phylogenies on the hypervariable regions of the 16S rRNA gene, six diverse groups could be identified, and their phylogenetic relationships retraced those of the complete 16S rRNA gene phylogeny (Figs. 7 and 10). Moreover, hypervariable region diversity analysis pointed out that “*Ca*. Pinguicoccus supinus” was usually a stand-alone sequence within its group, with the only exception of the V4–V6 phylogenetic tree having a single OTU associated to the endosymbiont sequence (Fig. 10). On the contrary, the other related *Puniceicoccaceae*, which were isolated and characterized from the environment (mostly marine), clustered in groups encompassing numerous OTUs (Fig. 10). The same stand-alone phenomenon occurred also for the epixenosomes, the aforementioned ectosymbionts of the ciliate *Euploditium arenarium*, that always formed a defined and separated lineage in all our analyses (Fig. 10). The limited presence of OTUs associated to these microorganisms could be also explained with the symbiotic nature of these bacteria, which occupy a peculiar and circumscribed ecological niche, possibly making their detection more complicated. It is also worth noticing that the number of OTUs *per* clade is significantly different when region V1–V2 is compared to region V4–V6 (Fig. 10). Indeed, in region V1–V2 “*Ca*. Pinguicoccus supinus” and related clades (in red) are relatively richer in OTUs if compared to region V4–V6, whereas the opposite is true for the *Coraliomargarita*-“*Fucophilus*”-*Puniceicoccus* clade (in green). This observation suggests that commonly used primers for region V1–V2 and V4–V6 could recognize the two clades with a different efficiency, producing artefactual results in terms of abundance and biodiversity.

### Proposal of an updated approach in taxonomy: the “next generation taxonomy”

The present work aims to propose and introduce a more comprehensive approach to the characterization of living beings, which integrates the holobiont concept according to Margulis^16^ into traditional and integrative taxonomy, leveraging the power of high-throughput DNA sequencing.

Starting from de Bary’s definition of symbiosis^4^, Lynn Margulis defined a holobiont as the compound of different species that together form a single ecological unit^16^. In recent times, an increasing interest regarding holobionts has arisen, leading to different interpretations of the concept^143,144^ and to different theories on the underlying evolutionary driving forces^143–149^. Nevertheless, the applicability of the “holobiont” definition, the evolutionary implications of the concept and, in particular, the *’boundaries’* of the holobiont, i.e. which associations should be considered within this definition, are still matters of debate.

Holobionts have been described among many and far-related groups of living beings, such as plants (for review see^150,151^), algae^152^, insects (for review see Refs.^153–155^), corals^156^ (for review see Refs.^157,158^), and even humans^159^. In this landscape, protists are under-represented, even though they are highly diverse and environmentally widespread^160–162^, and their association with a wide range of other microorganisms is well known^163–166^ (for review see Refs.^167–171^).

In our opinion, the application of the holobiont concept could be very useful for the characterization of living beings, introducing a new parameter for their taxonomic description, i.e. the presence of stably associated organisms, whenever present. Indeed, the presence of these organisms can influence the host, its physiology, and its adaptability to the environment^6,12,13^; moreover, in some cases it has been shown to influence also its morphology^5,14^, the fundamental parameter of traditional taxonomy.

The proposed approach aims to combine all the associated species characterizations into the holobiont description, providing not only morphological characters and molecular markers, but also functional and associative parameters. According to this line of thought, such a multidisciplinary approach represents a considerable improvement, allowed by the development of more sophisticated and powerful tools in molecular biology and genomics.

The whole mitochondrial genome, that we are proposing as a novel taxonomic descriptor for its amount of genetic information and the relative ease in sequencing and annotation, is becoming widely used in phylogenomics and evolutionary studies^172–174^. Indeed, the resolutive power of the mitogenome was employed to disentangle systematic issues^175–178^.

Although the number of articles treating mitogenomes are increasing, also leading to the birth of several dedicated online databases, such as ParameciumDB^179^, these analyses are still rarely used for a more comprehensive description of living beings, from the beginning (i.e. in their taxonomic description).

The aim of our proposal is, indeed, to fill this gap and, in parallel, to further promote the use of mitogenomes. This would increase the number of annotated mitogenomes of ciliates and other protists in online repositories, and therefore allow the development of dedicated databases.

To sum up, we suggest the inclusion of two additional descriptive parameters, i.e. the host's mitogenome and the characterization of the possibly present associated bionts, integrating novel useful features to the description of organisms. Proposed approach could be referred to as “next generation taxonomy”, to differenziate it from traditional “integrative taxonomy” that is not using Next Generation Sequencig data. It is to be emphasized that, applying the new approach by no mean implies that the traditional taxonomic tools are to be considered obsolete, as they are indisputably the base of species description.

To exemplify the concept of “next generation taxonomy” applied to holobionts, we present the ciliate *E. vanleeuwenhoeki* sp. nov. and its symbiont, “*Ca.* Pinguicoccus supinus” gen. nov., sp. nov., as a case study. In *E. vanleeuwenhoeki* sp. nov. a sole, stably associated bacterium was detected, namely the endosymbiont “*Ca*. Pinguicoccus supinus”, which makes the application of the holobiont concept to species description unambiguous. As detailed above, the peculiar features of this symbiont, especially the genomic ones, are strongly indicative of a long coevolutionary and adaptation history with its host, so that it might even be hypothesized that this bacterium might possibly represent an autapomorphy of the host species. Further study on different strain of the same species and on other related species will be necessary to assess this possibility.

Not by chance, it has already been demonstrated that some phylogenetically related *Euplotes* species showed the obligate symbiosis with certain bacteria (e.g. *P. necessarius*, “*Ca*. Protistibacter heckmannii”, “*Ca.* Devosia” spp.) as a peculiar evolutionary inherited trait^49,67,98,99,180,181^. Given the possible relevance of symbiotic events in *Euplotes* evolution and the extremely reduced genome of “*Ca.* Pinguicoccus supinus”, it seemed opportune to treat the sum of the different parts (*E. vanleeuwenhoeki* plus its symbiont) in a unitary and integrated way, starting from the very beginning, i.e. the taxonomic description”.

In conclusion, in our view, the proposed “next generation taxonomy” approach can constitute an updated and valuable standard for unicellular eukaryote descriptions and redescriptions. Indeed, integrating standard taxonomical analyses with the most modern tools in genomics and bioinformatics and the concept of holobiont, provides additional descriptors convenient not only to fully and unambiguously define a species but also to infer its interaction with other organisms and, at least partially, its biology. At a practical level, the suitability of the proposed approach for unicellular eukaryotic organisms, in particular free-living forms, associated with bacterial endobionts, is herein documented with the description of *E. vanleeuwenhoeki*, and we are rather confident that it should be similarly straightforward if applied to other kinds of holobiontic protists.

## Description of “*Candidatus* Pinguicoccus” gen. nov.

*Pinguicoccus* [Pin.gui.coc’cus. L. adj. *pinguis* fat; N.L. n. *coccus* (from Gr. masc. n. *kokkos* grain, seed) coccus: N.L. masc. n. *Pinguicoccus*, a fat coccus, because of its rounded body shape and because it was often found associated with host’s lipid droplets].

The genus description at present is the same as the description of the type species “*Ca.* Pinguicoccus supinus”.

## Description of “*Candidatus* Pinguicoccus supinus” sp. nov.

*Pinguicoccus supinus* (su.pi'nus. L. adj. *supinus* lying on its back, supine, indolent, lazy, because it is not a motile microorganism and because it lacks several fundamental metabolic pathways).

Type species of the genus. Non motile *Verrucomicrobia* bacterium of family *Puniceicoccaceae* (class *Opitutae*). Roundish-ovoid in shape, detected in the cytoplasm of the ciliate *Euplotes vanleeuwenhoeki,* with a diameter of 1.3–2.3 µm. It usually lies beneath the ciliate cortex, often in clusters of several individuals. Cells are delimited by a double membrane with a thin space between the two layers; no symbiosome has ever been observed. In several individuals, an invagination of the inner membrane and folding of the outer membrane are observed. Homogeneous bacterial cytoplasm. Sometimes shows presence of nucleoids. Reproduce by binary fission in the host cytoplasm, by means of an apparently typical symmetrical division. Its genome size is 163,218 bp and G + C content is 25%. The genetic code for protein translation is “translation table 4”.

Base of attribution: 16S rRNA gene sequence, MK569697; complete genome sequence CP039370; recognized by oligonucleotidic probes EUB338 VII and Pingui_1174.

## Experimental procedures

### Sample collection and cell culturing

The strain KKR18_Esm was collected on August 8th 2014, in one emissary of the freshwater Lake of Kolleru (16°36′05.0″N 81°18′47.8″E). Kolleru Lake is the largest freshwater body of India, and a protected Ramsar area due to seasonal migratory birds. It is situated between the deltas of two major rivers, Godavari and Krishna, 15 km south-east from the city of Eluru, in Andhra Pradesh state. The depth of Kolleru Lake is usually around 150–300 cm, but at the time of sampling, the lake was almost dried up and the water level had dropped to the depth of 60–90 cm^181^. Samples were collected at a depth of 15–30 cm near the shore of the lake using sterile 50 ml Falcon tubes: both water and sediment were collected at the same time^181^.

The original sample was screened by pouring about 20 ml of water in a Petri dish. Single cells were collected using a micropipette, washed several times in mineral water, put in a depression slide and enriched with a few drops of the monoclonal culture of *Dunaliella tertiolecta* (original salinity 5‰, diluted to freshwater) as food to obtain monoclonal cultures. These were maintained in an incubator at a temperature of 19 ± 1 °C and on a 12:12 h irradiance of 300 µmo photons/m^2^/s, and progressively adapted to 2.5‰ salinity by means of regular feeding (i.e. once a week) on *D. tertiolecta*.

### Live observations

Live ciliates were observed for morphological identification using a differential interference contrast (DIC) microscope with a Leitz Orthoplan microscope (Weitzlar, Germany), with the help of a compression device^182^ in order to distort them as little as possible. For examination of the swimming behaviour, ciliates were observed in a Petri dish, under a stereomicroscope (WILD HEERBRUGG, Switzerland)^181^.

### Silver and Feulgen stainings

Ciliates were treated for silver staining analysis with Champy’s solution and then with silver nitrate according to Ref.^183^, to stain the ciliary pattern. Feulgen staining procedure was performed to reveal the nuclear apparatus, using a protocol modified from Ref.^184^, after cell immobilization with celloidin-diethyl ether-alcohol solution^181^.

### Scanning electron microscopy (SEM)

The specimens were fixed in 2% OsO_4_ for 40 min and glued on small coverslips (snipped from 1 × 1 cm size to the size of stub) previously coated with Poly-L-Lysine and subjected to consecutive dehydration in an ethanol series. Samples were critical point dried according to Ref.^181^. Later, these coverslips were fixed onto SEM stubs with carbon conductive tape. Finally, the samples were sputter-coated with gold (Edwards sputter coater S 150B) and analyzed with JSM-5410 scanning electron microscope^181^.

### Transmission electron microscopy (TEM)

*Euplotes* cells were fixed in 2.5% glutaraldehyde in 0.1 M cacodylate buffer for 45 min, rinsed in 0.1 M cacodylate buffer and post-fixed in 1.5% aqueous osmium tetroxide in distilled water for 45 min at room temperature. Then cells were dehydrated and embedded in an Epon-araldite mixture as elsewhere described^32^. The blocks were sectioned with an RMC PowerTome X ultra-microtome. Sections were placed on copper grids and stained with uranyl acetate and lead citrate. Samples were visualized using a JEOL JEM-100SX electron microscope^181^.

### Measurements and recordings

Morphometric data of properly oriented cells were taken by using both live and stained specimen preparations (i.e. Feulgen, silver staining, SEM). Optical microscopy pictures were captured with a digital camera (Canon Power Shot S45) and used to obtain dimensions of living and stained ciliates. Morphometric measurements were analyzed with ImageJ 1.46r software^185^.

Based on micrographs of living and stained cells, accurate schematic line drawings were produced with a procedure described by Ref.^186^, which employed bitmap graphics with the GNU Image Manipulation Program (GIMP)^181^**.**

Terminology and systematics are mainly according to Ref.^187–189^.

### DNA extraction and 18S rRNA gene sequencing

Approximately 100–150 cells of KKR18_Esm strain were individually washed 3–5 times in sterile distilled water and fixed in 70% ethanol. Total genomic DNA extraction was performed using the NucleoSpin Plant II DNA extraction kit (Macherey–Nagel GmbH and Co., Düren NRW, Germany)^181^.

Polymerase chain reaction (PCR) was performed in a C1000 Thermal Cycler (Bio-Rad, Hercules, CA). The almost full-length of the 18S rRNA gene of *Euplotes* was amplified using the primer combination listed in Supplementary Table 7. High-fidelity Takara Ex Taq PCR reagents were employed (Takara Bio Inc., Otsu, Japan) according to the manufacturer’s instructions. PCR cycles were set as follows: 3 min 94 °C, 35 × [30 s 94 °C, 30 s 55 °C, 2 min 72 °C], 6 min 72 °C. PCR products were purified with the Eurogold Cycle-Pure Kit (EuroClone, Milan, Italy) and subsequently sent for direct sequencing to an external sequencing company (GATC Biotech AG, European Custom Sequencing Centre, Germany) by adding appropriate internal primers^181^ (see Supplementary Table 7).

### Whole genome amplification and assembly

Starting from around 5–10 cells, the total DNA material was amplified via the whole-genome amplification (WGA) method, using REPLI-g Single Cell Kit (QIAGEN, Hilden, Germany). The cells of KKR18_Esm strain were washed in distilled water for three times and the last time in PBS buffer. Then, they were transferred to a 0.2 ml eppendorf together with 4 µl of PBS. The WGA protocol was completed following the manufacturer’s instructions. This DNA material was processed with a Nextera XT library and sequenced at Admera Health (South Plainfield, USA), using Illumina HiSeq X technology to generate 75,510,798 reads (paired-ends 2 × 150 bp). Preliminary assembly of resulting reads was performed using SPAdes software (v 3.6.0)^190^.

### Mitochondrial genome assembly and annotation

Contigs representing the mitochondrial genome were identified using the Blobology pipeline^191^, and by tblastn searches using as queries proteins from reference genomes downloaded from NCBI, namely *Oxytricha trifallax* (JN383842) and *Euplotes minuta* (GQ903130). Contigs with a GC content comprised between 19 and 30%, and a coverage higher than 1000X were selected and a subset of the extracted reads were assembled with SPAdes. We decided to use approximately 10% of the extracted reads to artifically reduce the reads coverage, as coverage above 100 × generally produces worse assemblies, due to an increasing number of exactly replicated sequencing errors, which create false branches in the deBruijn graph. The assembled genome was annotated using PROKKA 1.10^192^, setting the DNA translation codon table “4” and then manually checked.

### Endosymbiont genome assembly and annotation

The presence of symbionts and host related microbial consortium was inspected in the preliminary assembly, using Barrnap^193^ to detect 16S rRNA gene sequences, and manually checking all the contigs annotated as bacterial. This, in conjunction with the use of the Blobology pipeline as indicated above, allowed contigs selection with a GC content lower than 25% and a coverage higher than 1000X for the assembly of the symbiont genome, and a subset (about 10% of the extracted reads. See mitochondrial genome assembly and annotation in “Experimental procedures” section) of the extracted reads were re-assembled with SPAdes. Closure of the circular genome was confirmed via PCR. Specific primers were designed on the basis of genome assembly and used for PCR amplification [Pingui_F162297 (5′—GTT GTA GCT CTC GGA TCG—3′), Pingui_R436 (5′—GTA GAG CAT CTT CGA CTC G—3′)]; the following internal primers were used for sequencing PCR products [Pingui_F163091 (5′—CTC AGA GCA CTC TGA GAT AG—3′); Pingui_R199 (5′—GTT TAG CTC TTC CGA GAT CG—3′)]. PCR cycles were set as follows: 3 min 94 °C, 35 × [30 s 94 °C, 30 s 55 °C, 2 min 72 °C], 6 min 72 °C. We relied on the same reagents, instruments and sequencing company cited above.

The assembled genome was annotated using PROKKA 1.10^192^, setting the DNA translation codon table “4” and then manually checking the results. The predicted protein-coding genes were also classified using NCBI COGs^194^, and compared with selected small genomes (Supplementary Table 8) and previously characterized *Verrucomicrobia* genomes (Supplementary Table 5). The COGs thus obtained were used to carry out a Principal Component Analysis (PCA), using SciPy packages in Python, taking into account the numerosity of each COGs class.

### Phylogenetic and phylogenomic analyses

The 18S rRNA gene of the host and the 16S rRNA gene of the newly characterized symbiont were aligned with the automatic aligner of the ARB software package version 5.5^195^ on the SSU ref NR99 SILVA database^196^. For the analysis on the host, 53 18S rRNA sequences of other members of the *Euplotes* genus, plus 7 sequences of other Spirotrichea as outgroup, were selected (dataset 1).

For the analysis on the symbiont, 98 16S rRNA sequences of other members of *Verrucomicrobia* were selected, plus 12 other sequences belonging to the superphylum *Planctomycetes, Verrucomicrobia, Chlamydiae* (PVC)^197^ as outgroup (dataset 2). Sequences not shown in the tree are listed in (Supplementary Table 9).

After manual editing to optimize base pairing in the predicted rRNA stem regions in each dataset, the two alignments were trimmed at both ends to the length of the shortest sequence. A positional filter was applied to dataset 1, to keep only those columns where the most conserved base was present in at least 10% of the sequences. Resulting matrices contained respectively 1843 (dataset 1) and 1456 (dataset 2) nucleotide columns, which were used for phylogeny and for the identity matrix calculation.

For each phylogenetic dataset, the optimal substitution model was selected with jModelTest 2.1^198^ according to the Akaike Information Criterion (AIC). Maximum likelihood (ML) trees were calculated with the PHYML software version 2.4^199^ from the ARB package, performing 1000 pseudo-replicates. Bayesian inference (BI) trees were inferred with MrBayes 3.2^200^, using three runs each with one cold and three heated Monte Carlo Markov chains, with a burn-in of 25%, iterating for 1,000,000 generations^181^.

For the phylogenomic analysis, 67 *Verrucomicrobia* (the endosymbiont plus 66 complete genomes from the genome taxonomy database (GTDB)^140^, and four other members of the PVC superphylum as outgroup, were used. A set of pre-aligned 120 single copy markers were employed^140^, and the “*Ca.* Pinguicoccus supinus” orthologs were identified by blastp search, then added to the existing alignments with MAFFT v7.123b^201^ and concatenated. A positional filter was applied to the concatenated genes according to Ref.^140^, to obtain a total of 34,747 sites. The best substitution model was estimated with ProtTest^202^. RaxML^203^ was used to estimate ML phylogeny with 1000 bootstraps.

### Fluorescence microscopy

*Euplotes* specimens were fixed for Fluorescence In Situ Hybridization (FISH) experiments in 2% OsO_4_ and dehydrated after fixation with an increasing ethanol series for 10 min each. Specimens were processed for hybridization experiments according to previous publications^181,204^.

Preliminary FISH experiments were carried out using the generic probe for *Verrucomicrobia* EUB338 III^205^ and the generic probe for *Bacteria* EUB338^206^. Only the EUB338 III probe showed a slightly positive signal.

After 16S rRNA sequencing, we designed two probes on the 16S rRNA gene of the putative endosymbiont of *Euplotes*, EUB338 VII (5′—CTG CTG CCA TCC GTA GAT GT—3′) and Pingui_1174 (5′—ACT GAC TTG ACG TCA TCC TCA—3′). Both probes were tested in silico on the Ribosomal Database Project (RDP)^207^ and SILVA database using TestProbe 3.0^196^, allowing 0 mismatches. The sequence of probe EUB338 VII matched 475 bacterial sequences in the RDP database, 19 of them registered as *Verrucomicrobia* and 139 registered as “unclassified bacteria”. While probe Pingui_1174 matched 220 bacterial sequences, 35 of them registered as *Verrucomicrobia* and 180 registered as “unclassified bacteria”. Sequences of these two new probes were deposited into probeBase database^208^. Those probes were used to perform additional FISH experiments to confirm the presence of that particular species of *Verrucomicrobia* inside the host.

Hybridized slide preparations were observed under a Zeiss AxioPlan fluorescence microscope (Carl Zeiss, Oberkochen, Germany) equipped with an HBO 100 W/2 mercuric vapor lamp at the following UV wavelengths: ~ 495 nm, ~ 550 nm. Digital images were captured at different magnifications (40X and 100X) by means of a dedicated software (ACT2U, version 1.0)^181^.

### Endosymbiont 16S rRNA gene screening on IMNGS

Diversity and environmental distribution of the bacterial endosymbiont was estimated using the IMNGS on-line platform^56^, which screens most of 16S rRNA gene amplicon datasets available. A query at 95% similarity was performed using the endosymbiont’s full-length 16S rRNA gene and related *Puniceicoccaceae* sequences (accession numbers: AB073978, AB372850, AB614893, AB826705, CP001998, DQ539046, EU462461, KT751307, JQ993599, JQ993517, Y19169, for their 16S rRNA full-length gene phylogenetic position see Fig. 7). The obtained sequences were longer than 300 bp and were divided into three different groups to perform phylogenetic analysis according to the 16S rRNA gene hypervariable regions V1–V2, V4–V6, and V7–V8. Sequences were clustered in OTUs with a 99% threshold similarity using UCLUST^209^, then they were aligned with MUSCLE^210^, and FastTree^211^ was employed to infer phylogenetic analyses. Thereafter, environmental distribution was investigated using the endosymbiont sequence as query and their abundances were calculated.

### Nomenclature acts

The present work has been registered in ZooBank (code: LSID:urn:lsid:zoobank.org:pub:231DF703-DBA8-4839-9755-5C71D6872406), as well as the new *Euplotes* species, *Euplotes vanleeuwenhoeki* (code: LSID:urn:lsid:zoobank.org:act:19C2249B-BF2D-4E81-9391-A271D38CD7B6). The correspondent web pages are available at the following addresses: https://www.zoobank.org/References/231DF703-DBA8-4839-9755-5C71D6872406 and https://www.zoobank.org/NomenclaturalActs/19C2249B-BF2D-4E81-9391-A271D38CD7B6 , respectively.

## Supplementary information

Supplementary Tables.

## Data Availability

All data generated or analysed during this study are included in this published article. All the 18S rRNA sequences, mitogenome sequence, “*Ca.* Pinguicoccus supinus” genome have been deposited in GenBank. Permanent slides are available from museums (indicated in the text) and from the corresponding author on reasonable request.
